# The Use of Convalescent Sera in Immune-Electron Microscopy to Detect Non-Suspected/New Viral Agents

**DOI:** 10.3390/v7052683

**Published:** 2015-05-22

**Authors:** Antonio Lavazza, Cristiana Tittarelli, Monica Cerioli

**Affiliations:** 1 Electron Microscopy Laboratory, Virology Department, Istituto Zooprofilattico Sperimentale della Lombardia e dell’ Emilia Romagna “Bruno Ubertini” (IZSLER), Via Bianchi 7/9, 25124 Brescia, Italy; 2 Istituto Zooprofilattico Sperimentale del Piemonte, Liguria e Valle d’Aosta, Sezione di Genova Piazza Borgo Pila 39, 16129 Genova, Italy; E-Mail: c.tittarelli-esterno@sanita.it; 3 Epidemiological Unit, Istituto Zooprofilattico Sperimentale della Lombardia e dell’Emilia Romagna “Bruno Ubertini”, Via Bianchi 7/9, 25124 Brescia, Italy; E-Mail: monicapierangela.cerioli@izsler.it

**Keywords:** negative staining, diagnosis, immuno-aggregation electron microscopy, viruses, animals

## Abstract

Negative staining electron microscopy methods can be employed for the diagnosis of viral particles in animal samples. In fact, negative staining electron microscopy methods are used to identify viruses, especially in minor species and wild animals, when no other methods are available and in cases of rare, emerging or re-emerging infections. In particular, immune-electron-microscopy with convalescent sera is employed to detect etiological agents when there are undiagnosed clinical outbreaks, when alternative diagnostic methods fail due to the lack of immunological reagents and primers, and when there is no indicative clinical suspect. An overview of immune-electron-microscopy with convalescent sera’s use in the diagnosis of new and unsuspected viruses in animals of domestic and wild species is provided through the descriptions of the following four diagnostic veterinary cases: (I) enteric viruses of pigs: Porcine Rotavirus, Porcine Epidemic Diarrhea Virus, Porcine Circovirus and Porcine Torovirus; (II) Rotavirus and astrovirus in young turkeys with enteritis; (III) Parvovirus-like particles in pheasants; and (IV) Lagoviruses: Rabbit Hemorrhagic Disease Virus and European Brown Hare Syndrome Virus.

## 1. Introduction

In the last thirty years, the use of negative staining electron microscopy methods (ns-EM), and immune-electron microscopy (IEM), has permitted the detection and identification of different viral particles in samples from several animal species. Compared with other diagnostic techniques, ns-EM excels in speed and “open view”, the ability to detect the “unexpected” without the need for specific reagents [1]. Therefore, ns-EM methods are very useful for identifying the causal agents as viruses when we are confronted with rare infections and new viruses, as well as for viral infections in minor species and wild animals, when no other standardized methods and/or diagnostic reagents are available [2,3,4].

The most relevant IEM method for diagnostic EM of viruses is immuno-aggregation electron microscopy (IAEM). Antibodies are mixed with virus suspensions to form differently sized aggregates of morphologically similar particles which are surrounded by a fuzzy halo of antibodies. In addition to the primary serum’s induction of aggregates and clumps of virions, a secondary step to improve the detection of virus-antibody complexes may be added. Electron-dense colloidal gold particles can be used to enhance (decorate) the visibility of antigen-antibody complexes. The combined techniques, commonly defined as IEM-gold methods, may employ as the “detector” either Protein A or G-gold conjugates that can bind the antibody heavy chains of several species or even secondary anti-species antibodies to which colloidal gold particles have been electrostatically adsorbed [5,6].

IAEM is more specific than other ns-EM methods, combining a morphological identification with antigen specificity, and it is more sensitive, making the identification of known and unknown viruses possible and easier, as well as improving the ability to locate viruses, especially under one of the following conditions: (a) when virions are pleomorphic and difficult to identify because they do not have a typical viral morphology, such as defined symmetry, presence and shape of spikes, particle size, and/or number and arrangement of capsomers; (b) when the viral concentration is low (this technique can provide a 10^2^–10^3^ enrichment [2]); or (c) when the samples are “dirty” because the aggregated complexes are more easily observed.

IAEM may employ, as primary antibody, hyperimmune sera, produced in the same species or in heterologous species, or monoclonal antibodies. Additionally, convalescent sera can also be used. According to Hazelton and Gelderblom [4], convalescent phase sera collected from a case-patients (single surviving animal or animals from the same herd/flock/group, in the case of outbreaks occurring in farm animals) 4–6 weeks after the onset of illness are powerful diagnostic reagents. If no agent has been identified by standard detection procedures, such as ns-EM, tissue culture, immunoassay or nucleic acid amplification techniques, these serum samples may be used to improve the sensitivity of the EM method and to detect the causal agent.

Our objective is to provide an overview of the use of IAEM with convalescent sera for the diagnosis of new and unsuspected viruses in animals of domestic and wild species by describing four diagnostic veterinary cases.

## 2. Materials and Methods

### 2.1. Preparation of Convalescent Sera

Convalescent sera are normally taken 20–30 days after clinical signs are observed from a variable number of animals. Serum can be taken from a single animal or pooled from a group of up to 20 animals, based on the species and size of the group/herd/flock. The pooled sera are centrifuged at 12,800× *g* for 30 min, inactivated by heating at 56 °C for 30 min and passed through 22 µm filters. Prior to use, convalescent sera are examined using standard direct ns-EM to ensure that they are free from viral particles, and lipid and protein debris. Grids are prepared using the Airfuge method (see Section 2.2 for details) and defined as clean (“blank”), after random screening of the grid for 15 min (at least 5 grid squares).

#### Determination of the Optimal Dilution of Sera

To achieve good results using the IAEM method, and particularly to induce a proper aggregation/precipitation of virions, it is essential that virions and antibodies are at equal concentrations (“zone phenomenon”) [7]. The dilution is optimal when the immunoaggregates are of adequate size, the halo of antibodies around the virions is not too thick and the virion’s morphology is not altered. To ascertain the optimal dilution, one sample is tested using serial dilutions of the pooled convalescent sera. Initially, 1:2, 1:20, 1:200 and 1:1000 dilutions in phosphate buffer saline (PBS) are tested, and then, based on the results obtained, appropriate intermediate dilutions (1:10, 1:50, 1:100, 1:500, *etc.*) are used. The determination of the optimal dilution is visual. The observer determines the best combination of aggregate size, antibody halo and virion morphological integrity.

### 2.2. IAEM Method

Different types of samples, either taken *in vivo*, such as feces, fluids or swabs, or *post-mortem* during necropsy, such as viscera or organ contents, may be examined by ns-IAEM. In the first case, the convalescent sera may be taken from the same clinically affected animals, while in the second case, animals of the same group/herd/flock are sampled. Fecal materials and fluids are diluted 1:10 (*v*/*v*) in sterile distilled water. Swabs (ocular, nasal, tracheal, fecal *etc.*) are re-suspended in 1 mL sterile distilled water. Organs (spleen, liver, kidney, lung *etc.*) extracts (1:10 *w*/*v* in sterile distilled water) are obtained by mechanical homogenization using Ultraturrax (IKA^®^-Werke GmbH & Co. KG, Staufen, Germany).

The samples are first frozen, thawed twice, and then centrifuged at 5500*× g* for 20 min and at 12,800*× g* for 10 min for clarification. The second supernatant (85 µL) is mixed with the same amount of each serial dilution of the convalescent sera and then incubated for 1 h at 37 °C before ultracentrifugation using an Airfuge (Beckman Instruments, Palo Alto, CA, USA) for 15 min at 21 psi (82,000*× g*). The Airfuge is fitted with an A-100/18 rotor holding six 175 µL test tubes containing specific hand-made adapters with 3 mm grids, which allow direct pelleting of viral particles onto a glow-discharged EM sample support (300 mesh copper grid, covered with a carbon reinforced plastic film). The adapters are almost identical to those provided in the past by Beckman. They are made of Plexiglas and shaped to fit perfectly into the tubes. The 3-mm grids are put in a slot obtained on the face of the adapter which has a slant cut with the same angle of the rotor (18°) in order to permit direct pelleting of particles on their surface. Grids are then stained using 2% sodium phosphotungstate (NaPT; Fluka Chemie AG, Buchs, Switzerland), pH 6.8, for 1.5 min and observed with a TEM operating at 80–85 kV, 30,000×. At IZSLER, a Siemens Elmiskop 102 TEM was used till 1988, from 1988 to 2011 a Philips CM10 TEM was used and since August 2011 a FEI Tecnai G2 Spirit Biotwin (FEI, Eindhoven, The Netherlands) TEM was used.

### 2.3. Controls

The controls employed to ascertain the correctness and accuracy of the IAEM reaction include: (I) preparation of the sample with no serum; (II) incubation with a pre-serum (if available); and/or (III) incubation with another serum. Both negative control sera were used at fixed dilutions of 1:5 and 1:50. Basically, the use of controls at fixed dilutions is a compromise. In the execution of routine diagnostic IAEM tests, negative controls are used with the aim of excluding spontaneous aggregations of virions. The assumption is that both of the employed negative sera will not aggregate all of the virions potentially present in the sample. Therefore, their use at fixed dilutions is conventionally accepted because it is not the intent to detect and evaluate real differences in the levels of binding. They can, therefore, be considered as qualitative and not quantitative controls*.*

## 3. Results and Discussion

Some field cases of diagnosing animal diseases are representative of the results obtained by IAEM using convalescent sera to detect “new” and emerging viruses, but also mixed viral infections and non-cultivable viruses. In the subsequent paragraphs we describe the detection of viruses in clinical outbreaks pertaining to different species: enteritis in pigs and turkeys, and hepatitis in pheasant, rabbits and hares.

### 3.1. Porcine Rotavirus (PoRV), Porcine Circovirus (PCV2), Porcine Torovirus (PoToV) and Porcine Epidemic Diarrhea Coronavirus (PEDV) in Pigs with Enteritis

#### 3.1.1. Porcine Rotavirus (PoRV)

Rotaviruses (RVs) are the major etiological agents of viral enteric diseases in the young individuals of several mammalian and avian species [8]. RVs are classified into eight Groups (A–H) based on the nucleotide sequence identities of the VP6 gene, of which four (A–C and E) affect swine. Porcine rotaviruses (PoRVs) are the primary agents of enteritis in post-weaning pigs. They have a steady incidence in pig commercial farms and can sporadically cause enteritis outbreaks with increased mortality rates. The disease may be complicated by other pathogens, including enteric viruses, some of which have only recently been discovered, and with undefined pathogenic viruses, such as calicivirus [9,10,11], enterovirus, teschovirus, astrovirus [10], parvovirus, sapovirus [12], norovirus, bocavirus and kobuvirus.

**Figure 1:**
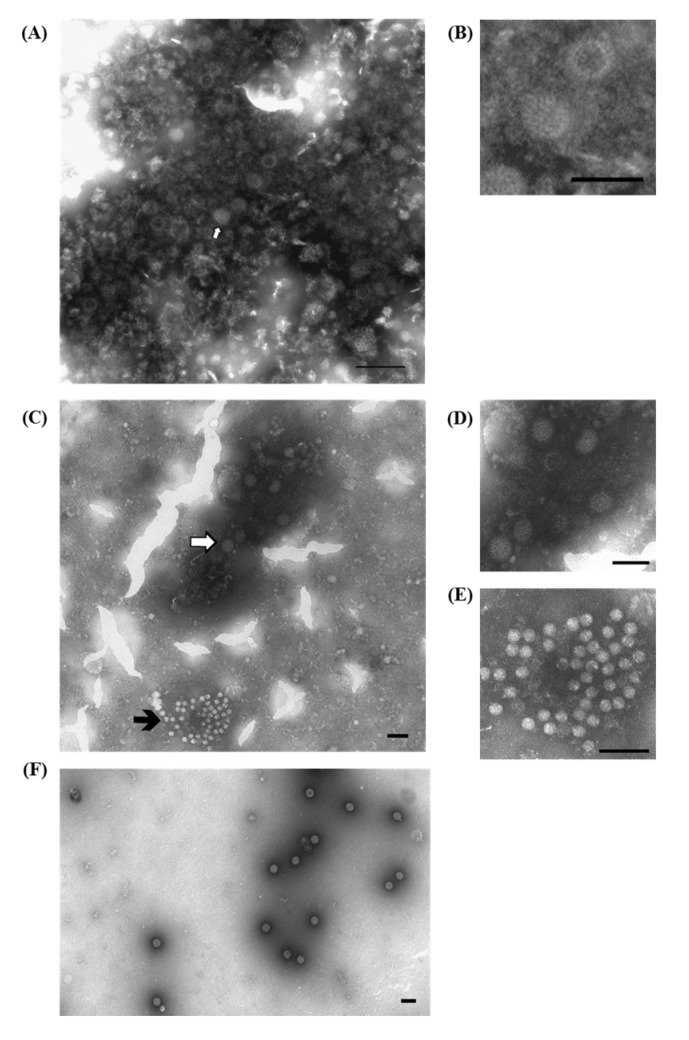
Electron micrograph of ultracentrifuged fecal contents from pigs with post-weaning enteritis. (**A**) A group of rotavirus particles (⇧) with a dense halo of antibodies showing different levels of morphological integrity is visible; (**B**) Higher magnification of (**A**) showing two rotavirus particles with the typical inner shell, which in one case is penetrated by the negative stain (empty particle). Immuno-aggregation electron microscopy (IAEM) preparation with convalescent sera diluted 1:80. Negative staining (2% sodium phosphotungstate). TEM FEI Tecnai G2 Spirit, 85 kV. Bar = 200 nm; (**C**) A large group of rotavirus particles (⇧) and a small group of small round fecal virus/enterovirus-like 28–30 nm particles (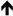) are visible; (**D**,**E**) Higher magnifications of (**C**) showing respectively one group of rotavirus and one group of small round fecal virus/enterovirus-like virus. IAEM preparation with convalescent sera diluted 1:80. Negative staining (2% sodium phosphotungstate). TEM Philips CM10, 80 kV. Bar = 100 nm; (**F**) IAEM negative control. Dispersed rotavirus particles. TEM Philips CM10, 80 kV. Bar = 100 nm.

A variety of laboratory techniques are available to establish a firm diagnosis of Group-A PoRVs, including ns-EM methods, fluorescent antibody techniques, immunohistochemistry, ELISA tests and PCR, which are also used for group-specific typing. The detection of non-A, atypical RVs can be difficult due to the usually lower intestinal load, the lack of specific reagents for immune-enzymatic tests, and the fact that characteristic single—and double—shelled particles may not be readily apparent during ns-EM observation [13]. Using IAEM with convalescent sera makes it possible to detect different PoRVs (Figure 1A–D), including those strains belonging to non-A RV groups, which are non-cultivable and not recognized by immuno-enzymatic tests based on Group-A reagents. Actually, only PCR amplification of targeted sequences using Group-specific oligonucleotide primers, followed by nucleotide sequencing, provides an alternative tool to identify non-Group-A RVs [11]. In addition, IAEM with convalescent sera more easily detects other viral agents, present in association with RVs, among which, in our experience [14], small round fecal virus/enterovirus-like particles are the most frequently present (Table 1). An example of a mixed infection in which RVs are associated with unclassified, 28–30 nm roundish particles, is shown in Figure 1C,E). IAEM negative control showing several rotavirus particles dispersed in the observation field is reported in Figure 1F.

**Table 1:** Results of IAEM examination of fecal or intestinal samples from pigs with enteric disease (2002–2007). The category “not classified” includes “enterovirus-like viruses” and the so-called “small round fecal viruses”. The numbers in italics indicate the percentages calculated based on the total examinations performed each year. The percentage of the positives exams is based on the total examinations although some examinations resulted in more than one positive identification due to the occurrence of multiple infections with two or more viruses in association.

Year	2002		2003		2004		2005		2006		2007		Total	
No. exams	716		788		622		384		746		333		3589	
No. negative	407	*56.8*	399	*50.6*	412	*66.2*	288	*75.0*	489	*65.5*	220	*66.1*	2215	*61.7*
No. positive	309	*43.2*	389	*49.4*	210	*33.8*	96	*25.0*	257	*34,5*	113	*33.9*	1374	*38.3*
Adenovirus	3	*0.4*	0	*0.0*	0	*0.0*	0	*0.0*	0	*0.0*	1	*0.3*	4	*0.1*
Calicivirus	3	*0.4*	2	*0.3*	6	*1.0*	0	*0.0*	5	*0.7*	3	*0.9*	19	*0.5*
Coronavirus	152	*21.2*	224	*28.4*	71	*11.4*	38	*9.9*	152	*20.4*	33	*9.9*	670	*18.7*
Circovirus	54	*7.5*	107	*13.6*	0	*0.0*	0	*0.0*	2	*0.3*	3	*0.9*	166	*4.6*
Enterovirus	16	*2.2*	2	*0.3*	0	*0.0*	0	*0.0*	15	*2.0*	19	*5.7*	52	*1.4*
Parvovirus	2	*0.3*	0	*0.0*	0	*0.0*	0	*0.0*	0	*0.0*	0	*0.0*	2	*0.1*
Rotavirus	59	*8.2*	99	*12.6*	123	*19.8*	40	*10.4*	56	*7.5*	55	*16.5*	432	*12.0*
Torovirus	4	*0.6*	0	*0.0*	0	*0.0*	0	*0.0*	0	*0.0*	0	*0.0*	4	*0.1*
Not classified	155	*21.6*	245	*31.1*	43	*6.9*	31	*8.1*	32	*4.3*	12	*3.6*	518	*14.4*

IAEM has been routinely used to diagnose enteric viruses in fecal or intestinal samples from pigs with enteric disease (Table 1). In northern Italy, from 2002 to 2007, 1374 (38.3%) out of 3589 IAEM examinations tested positive for one or more viruses [14]. Rotavirus (12%) and PED coronaviruses (18.7%) represented the most common viral agents. Other agents included circovirus (4.6%), enterovirus (1.4%), calicivirus (0.5%), adenovirus (0.1%), parvovirus (0.1%), torovirus (0.1%) and small round fecal viruses/enterovirus-like viruses (14.4%), which were not fully classified due to their undefined morphology [14]. Therefore, to improve the knowledge of unclassified enterovirus-like particles, a total of 40 viral isolates were further identified by sequencing the capsid VP1-encoding gene. This lead to their classification as porcine enterovirus, porcine teschovirus and porcine sapelovirus [15].

The comparison of IAEM results with Group-A ELISA tests and PCR methods indicated that (a) many RV strains belonged to non-A groups; (b) many isolates had previously remained undetected and had a high degree of genomic variability [11,16,17]; (c) even some Group-A RVs could not be detected using standard PCR methods [17]; and (d) PoRVs belonging to different groups (A, C and non-A/non-C) were often associated [11]. Moreover, the extent of multiple infections in enteric diseases raised diagnostic issues, and, in this regard, IAEM observation or the parallel detection of viral antigens/genomes in a multiplex format should be the most appropriate approach to obtain the correct laboratory diagnoses of enteric infections [11].

#### 3.1.2. Porcine Circovirus Type 2 (PCV2)

**Figure 2:**
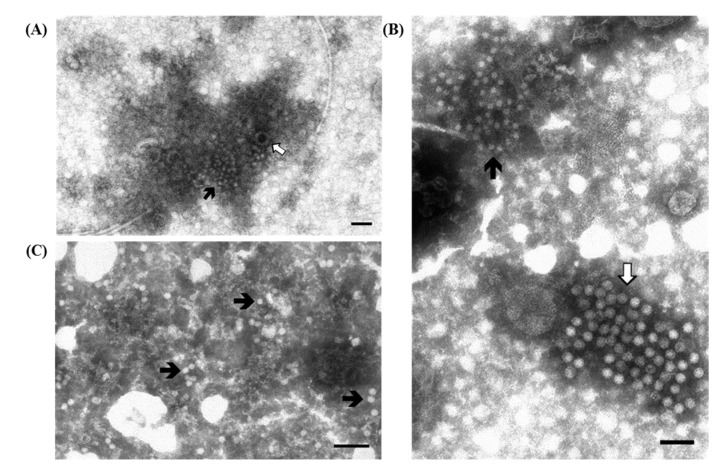
Electron micrographs of ultracentrifuged fecal contents from pigs with post-weaning enteritis. Small 18–20 nm virions resembling circovirus (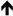) are grouped together with (**A**) a few partially destroyed and empty rotavirus particles (⇧) and (**B**) a large group of enterovirus-like particles (⇧). Immuno-aggregation electron microscopy preparation with convalescent sera diluted 1:40; (**C**) IAEM negative control. Several circovirus particles (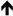) are visible, singularly distributed or in small groups. Negative staining (2% sodium phosphotungstate). TEM Philips CM10, 80 kV. Bar = 100 nm.

As reported before (Table 1) one of the viruses associated with PoRV is PCV2 (Figure 2A). PCV2 was initially isolated in 1991, was later associated with the Post-weaning Multisystemic Wasting Syndrome (PMWS), and it is still present with a high prevalence in commercial pig farms.

The small size (18–20 nm) of circovirus makes them difficult to recognize, especially when present as single dispersed particles in the background fecal material, and thus, they are more clearly observed in the intestinal contents of piglets when precipitated in aggregates by IAEM. PCV2 has an immuno-suppressive effect and, thus, it is often detected in association with other viral and bacterial agents. Thus, the use of IAEM with convalescent sera is useful for more readily identifying multiple viral infections in which PCV2 is associated with other viruses. In fact, in one study [18] we used IAEM with anti-PCV2 convalescent sera to examine 101 animals taken from 29 industrial farms where outbreaks of PMWS were reported or lesions typical of PMWS were regularly shown at necropsy. Prior to being used in the IAEM method, the convalescent serum used was determined to be positive (titer 1:10,000) for anti-PCRV2 antibodies by testing in an in-home MAb-based competitive serological ELISA test [19]. Forty-five samples tested positive for PCV2, and it was frequently observed in association with one or more other viruses (Table 2). Examples of the association of circovirus particles with other virions, and particularly with rotavirus and enterovirus-like virus particles, are respectively reported in Figure 2A,B.

**Table 2:** Types of viruses observed by EM and the frequency of cases in association with PCV2. The percentages are calculated based on the total number of cases with multiple infections (*n* = 45).

Viral Agent	No. PCV2 Pos.	%
**Coronavirus/PED**	27	60.0
**Enterovirus-Like**	26	57.8
**Rotavirus**	4	8.9
**Others (parvovirus, calicivirus, adenovirus)**	7	15.6

We then compared the results obtained with IAEM and two others virological methods, immunofluorescence (IF) and PCR, in the identification of PCV2 in the organs of clinically affected pigs sampled during outbreaks of PMWS [20]. Examinations tested different organs, such as lymph nodes and lungs using IF and PCR, and intestinal tracts using IAEM. The results confirmed the lower sensitivity of IAEM compared with PCR (concordance 44.1%) but not compared with IF (concordance 64.7%). By performing a single IAEM test, different associating particles, such as parvovirus-like, enterovirus-like, rotaviruses and PEDV, were simultaneously observed, thus more data were acquired on the interactions between PCV2 and other pathogens potentially involved in the pathogenesis of PMWS syndrome. Indeed, a higher level of PCV2 particles were detected in the caecum in comparison with the small intestine, and in several occasions only the caecum contained circovirus particles [20].

#### 3.1.3. Porcine Torovirus (PToV)

Another virus that we occasionally found in diarrheic post-weaned piglets, mostly associated with rotavirus, is Porcine Torovirus (PToV). The toroviruses are positive-stranded RNA, enveloped viruses, belonging to the family Coronaviridae. They have a typical morphology (discoid, kidney—and rod-shaped) and cause a serious, at times fatal, diarrheal disease in horses (Berna virus; BEV), cattle (Breda virus; BRV), gastroenteritis in humans and enteric infections in swine.

**Figure 3:**
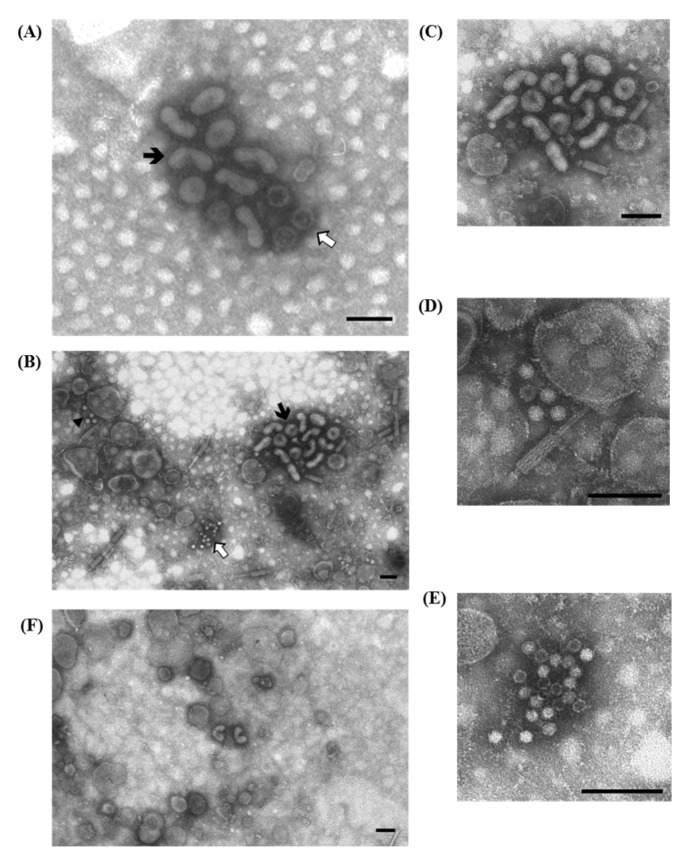
Electron micrographs of ultracentrifuged fecal contents from pigs with post-weaning enteritis. Torovirus “kidney-shaped” particles (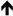) are grouped together with (**A**) rotavirus particles (⇧) and (**B**) groups of either enterovirus-like (▲) or circovirus (⇧) particles; (**C**) Higher magnification of (**B**) showing the aggregate of pleomorphic torovirus particles with a fringed surface in which short peplomers are immersed in an halo of antibodies; (**D**) Higher magnification of the small aggregate in the upper left of (**B**) composed by four full and one empty enterovirus-like particles close to a rod shaped phage-tail; (**E**) Higher magnification of (**B**) showing the group of empty and full circovirus particles; (**F**) IAEM negative control: two kidney-shaped torovirus particles are visible in the middle together with several isolated fringed particles. Immuno-aggregation electron microscopy preparation with convalescent sera diluted 1:80. Negative staining (2% sodium phosphotungstate). TEM Philips CM10, 80 kV. Bar = 100 nm.

After the first detection in 1990, using ns-EM, of torovirus particles in association with RVs in the feces of piglets with diarrhea (Figure 3A) [21,22], a convalescent serum was taken and its specificity was assessed by the immunoaggregation of particles molecularly characterized as PToV [22] and by serological comparisons with both field sera and anti-PToV, as well as anti-BEV hyperimmune sera [23]. The convalescent serum 233/90 [23] has since then been systematically used in IAEM in suspected cases, and in this manner six more cases of PToV were identified using IAEM: one each in 1996 and 1999, and four in 2002 [14], mostly in association with RVs and other enteric viruses (Figure 3B–E). Particles identified in these cases were further characterized by molecular methods [22,23] and one sample from 2002 was defined as a new PToV strain [23].

Considering the low frequency of PToV in diarrheic pigs, pre-selection by IAEM or pre-screening by diagnostic RT-PCR of ToV-positive fecal specimens could allow detailed studies of the genetic diversity among ToVs [22].

#### 3.1.4 Porcine Epidemic Diarrhea Virus (PEDV)

PEDV is a coronavirus that can infect and cause enteritis having typical clinical symptoms, including vomiting, diarrhea and dehydration, in pigs of all ages, but it is usually only fatal in piglets.

**Figure 4:**
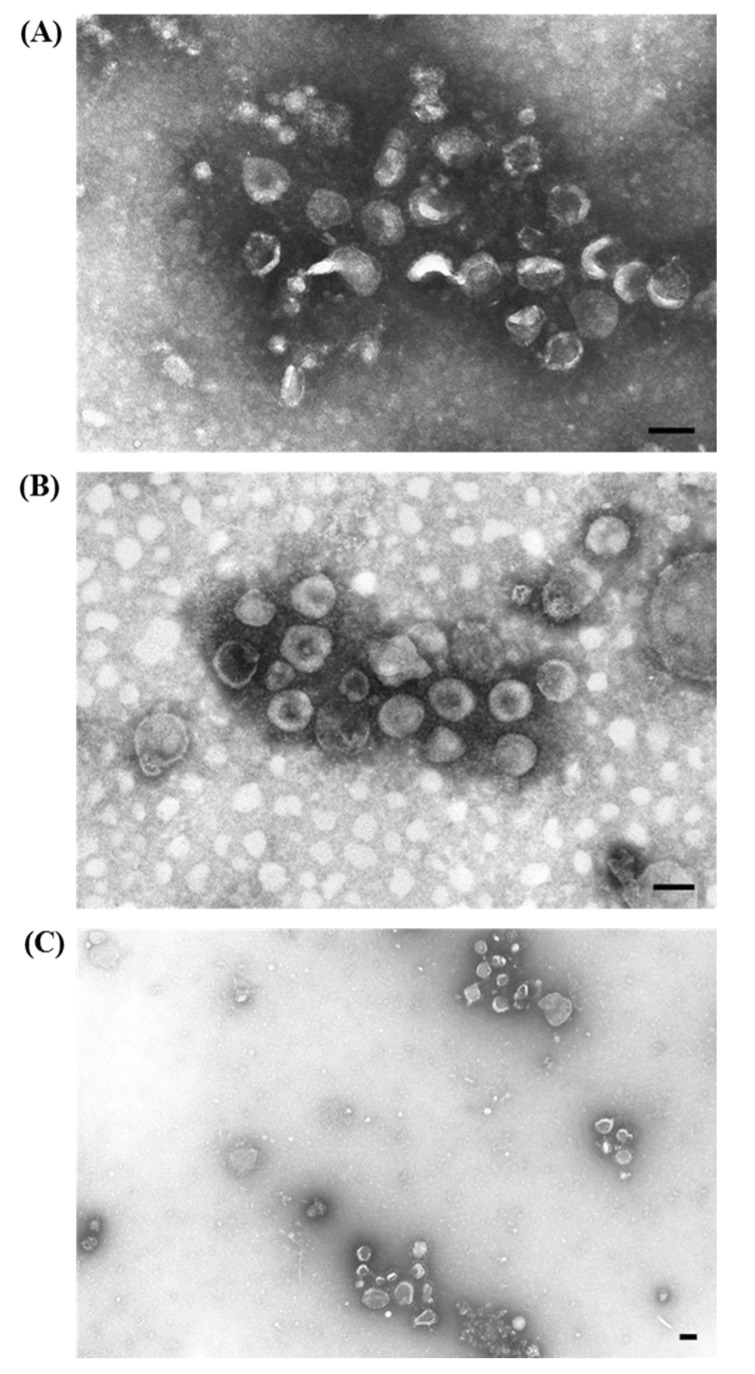
Electron micrographs of ultracentrifuged fecal contents from pigs with diarrhea from two distinct outbreaks (**A**,**B**). Coronavirus particles attributed to Porcine Epidemic Diarrhea virus (PEDV) are visible as aggregates. In both cases the particles are showing an external surface with spikes still visible but immersed in a halo of antibodies; (**C**) IAEM negative control: small groups of coronavirus particles with well–conserved surface spikes are visible. Immuno-aggregation electron microscopy with anti-PEDV convalescent sera diluted 1:20. Negative staining (2% sodium phosphotungstate). TEM Philips CM10, 80 kV. Bar = 100 nm

In Italy, PEDV was first identified by ns-EM in the early 1990s when a severe wave of PED outbreaks occurred. Thereafter, convalescent sera were taken and used to perform IAEM. In fact, PED was difficult to diagnose since the virus is hard to isolate *in vitro* and, at that time, few reagents and established methods were available. The specific aggregation by IAEM with convalescent sera of single dispersed fringed particles permitted better recognition and identified PEDV particles by their typical coronavirus morphology (Figure 4A–C). The specificity of the convalescent sera employed was ascertained using an immunoperoxidase monolayer assay with the Vero cell-adapted Belgian PEDV isolate CV777 as the antigen.

Then, PED became endemic with sporadic outbreaks occurring between cyclic epidemic peaks. From 1994 to 2000, 296 out of 2072 samples tested positive using IAEM. When the last epidemic wave occurred in 2005–2006 in the areas with dense pig populations in northern Italy [24], diagnostic methods were set up as screening tools to evaluate the presence and circulation of PEDV in field surveys. Two immunoassays, based on MAbs produced against the European CV777 reference strain were established for the detection of both antigen (double antibody sandwich ELISA) [25] and antibodies (MAb-based antigen capture ELISA) [26]. To confirm the virological identification we used reverse transcriptase-PCR (RT-PCR), as described by Kim *et al.* [27] that employs primers targeting the S gene [25]. From 2008 to 2014, only sporadic outbreaks were observed in grower and finisher herds. Using IAEM and sandwich ELISAs, 73 PEDV cases, from 60 different farms, out of 1756 cases of enteritis (4.54%) were diagnosed, and 28 strains were further genetically analyzed [28].

Since January 2015, Italy is facing a new important epidemic wave of PED caused by a strain similar to the USA OH851 mild strain (Lavazza A. and Boniotti B., personal observations). The practical approach for controlling PED is mainly based on active surveillance and on quick diagnoses using ELISA and PCR of fecal materials, as well as by a serological survey of the representative number of pigs on the farm (Alborali G.L. and Lavazza A. personal observations).

### 3.2. Rotavirus and Astrovirus in Young Turkeys with Enteritis

Rotaviruses (RVs) are the major etiological agents of enteric viral diseases in young individuals of several mammalian and avian species [29,30]. In turkey, RVs are often associated with astrovirus (AstV) [31] and cause, with a synergistic effect, clinical enteritis ranging from subclinical to severe [32]. Because of the antigenic variability of turkey RVs, which belong to different groups (A, D, F, and G), the inability to cultivate *in vitro* both AstV and non-A RVs, and the scarcity of established molecular techniques, EM methods are still useful to quickly achieve a diagnosis of viral enteritis. In particular, IAEM with convalescent sera could simplify and improve viral detection of RVs and AstVs by concentrating and increasing the number of visible particles through their aggregation to form immunocomplexes. Thus, IAEM with convalescent sera has been extensively used for diagnostic examinations of viral enteric infections.

**Figure 5:**
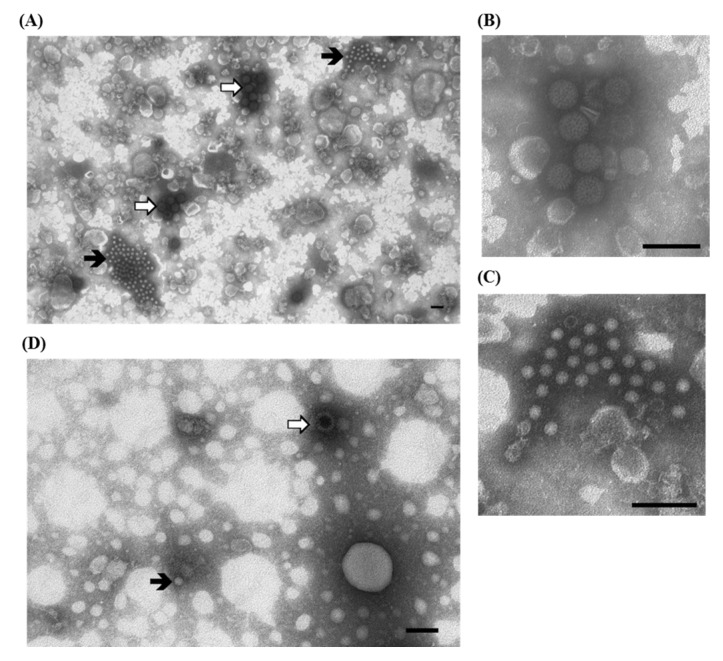
Electron micrographs of ultracentrifuged gut contents from 12—to 15-day-old turkeys. (**A**) Aggregates of both larger, roundish, 60–70 nm rotaviruses (⇧) and smaller, icosahedral non-enveloped 25–27 nm astroviruses (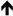), surrounded by a fuzzy halo of antibodies are visible; (**B**) Higher magnification of rotavirus particles: the presence of a halo of antibodies does not mask the typical single—and double-shelled morphology of rotaviruses; (**C**) Higher magnification of astrovirus particles: the surface of aggregated virions, even if not always distinctively showing the 5–6 star-shaped pattern, do not have a completely homogeneous or featureless surface, which is highly indicative of astroviruses; (**D**) IAEM negative control: one isolated rotavirus (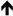) and one astrovirus-like particle (⇧) are visible. Immuno-aggregation electron microscopy (IAEM) preparation with convalescent sera diluted 1:40. Negative staining (2% sodium phosphotungstate). TEM Philips CM10, 80 kV. Bar = 100 nm.

During the period of 1994–2004, 481 fecal and gut samples derived from outbreaks of enteritis in commercial turkeys, which occurred mostly in northern Italy, were conferred to the laboratory and examined by IAEM [33]. All of the birds sampled were between 1 and 5 weeks old and were suffering from systemic and enteric symptoms characterized by growth retardation, lethargy, anorexia, dehydration, wet droppings and/or diarrhea, which led to weight loss, decreased flock uniformity and increased mortality. A pool of sera, taken three weeks after a clinical episode of enteritis caused by RVs and AstVs, was obtained from convalescent turkeys and used to perform IAEM. The results are reported in Table 3. Viral particles were observed in 51.4% of the examined samples and rotavirus (26.6%) and astrovirus (25.8%) mostly clumped in medium-large aggregates, were the most frequently detected particles (Figure 5A). The presence of antibody halos did not hamper the identification of virions, particularly of rotavirus, which, at the serum dilution used, were mostly showing a typical morphology (Figure 5B). AstVs were identified when the 28–30 nm picornavirus-like icosahedral particles displayed the characteristic 5–6 pointed star-shaped surface. However, since the typical star-shaped configuration is usually visible in only approximately 10%–20% of the particles [34], and it may sometimes be obscured by antibodies when examining stools by IAEM [35], we classified particles of this size-class as AstV if they showed a somehow structured surface (Figure 5C).

**Table 3:** Distribution of viral positivity by year and type of virus in fecal samples of 1–5 week old turkey poults showing enteritis. The numbers in italics indicate the percentages calculated based on the total examinations performed each year. The percentage of the positives exams is based on the total examinations although some examinations resulted in more than one positive identification due to the occurrence of multiple infections with two or more viruses in association.

Year	No.	Negative		Rotavirus		Astrovirus		Enterovirus-Like Virus		Parvovirus-Like Virus		Adenovirus		RSVLP		Coronavirus	
		No.	%	No.	%	No.	%	No.	%	No.	%	No.	%	No.	%	No.	%
1994	14	12	*85.7*	1	*7.1*	0	*0.0*	1	*7.1*	0	*0.0*	0	*0.0*	0	*0.0*	0	*0.0*
1995	11	1	*9.1*	3	*27.3*	4	*36.4*	3	*27.3*	2	*18.2*	0	*0.0*	0	*0.0*	0	*0.0*
1996	7	1	*14.3*	0	*0.0*	3	*42.9*	3	*42.9*	0	*0.0*	0	*0.0*	0	*0.0*	0	*0.0*
1997	26	17	*65.4*	7	*26.9*	2	*7.7*	2	*7.7*	0	*0.0*	0	*0.0*	0	*0.0*	0	*0.0*
1998	49	28	*57.1*	10	*20.4*	16	*32.7*	3	*6.1*	1	*2.0*	0	*0.0*	0	*0.0*	0	*0.0*
1999	48	23	*47.9*	11	*22.9*	13	*27.1*	2	*4.2*	0	*0.0*	2	*4.2*	0	*0.0*	4	*8.3*
2000	41	19	*46.3*	14	*34.1*	10	*24.4*	0	*0.0*	4	*9.8*	1	*2.4*	2	*4.9*	1	*2.4*
2001	64	26	*40.6*	22	*34.4*	14	*21.9*	12	*18.8*	2	*3.1*	0	*0.0*	0	*0.0*	0	*0.0*
2002	77	46	*59.7*	11	*14.3*	17	*22.1*	0	*0.0*	6	*7.8*	2	*2.6*	1	*1.3*	1	*1.3*
2003	118	50	*42.4*	43	*36.4*	32	*27.1*	6	*5.1*	6	*5.1*	3	*2.5*	0	*0.0*	4	*3.4*
2004	26	11	*42.3*	6	*23.1*	13	*50.0*	1	*3.8*	0	*0.0*	1	*3.8*	0	*0.0*	0	*0.0*
Total	481	234	*48.6*	128	*26.6*	124	*25.8*	33	*6.9*	21	*4.4*	9	*1.9*	3	*0.6*	10	*2.1*

During the observation of samples prepared using the IAEM method, we also identified other virions, which were sparsely distributed or even in small groups, but were not surrounded or immersed in a halo of antibodies. These included 6.9% enterovirus-like particles (possibly AstVs displaying an undefined morphology and a featureless surface), 4.4% of 18–22 nm parvovirus-like particles, 2.1% coronaviruses (the diagnosis was, in this case confirmed using a hyperimmune serum against turkey coronavirus, kindly provided by Prof. Mo Saif, Ohio State University, Wooster, OH, USA), 1.9% adenovirus and 0.6% rod-shaped virus-like particles (RSVLP) [36]. Often (79 cases), we simultaneously observed in the same sample two or more different viruses in a mixed infection. The combination of RVs plus AstVs was the most common (57 cases), supporting their pathogenic roles and importance as primary agents of enteritis (Table 4).

To better characterize and identify those particles observed using ns-EM methods that were morphologically classified as small round viruses (SRVs), AstVs and enterovirus-like viruses (ELVs), we further analyzed samples of different avian commercial species, including turkey, with RT-PCR targeting the open reading frame (ORF)-1b of AstV, which encodes an RNA-dependent polymerase. We then sequenced and genetically analyzed the RT-PCR positive samples, and we performed a phylogenetic analysis to distinguish and type them [37]. This study also permitted us to compare the results obtained by the two diagnostic methods (RT-PCR *vs*. ns-EM) and to draw some conclusions regarding their respective advantages and disadvantages. ns-EM was confirmed as a very useful method for an initial diagnosis of viral enteritis and for the identification and screening of RVs, SRVs, AstVs and ELVs in avian intestinal and fecal samples because it is fast, can identify viruses that cannot be isolated or identified using other methods, and is also able to distinguish mixed infections and particles that are unable to replicate. Nevertheless, it is only sensitive enough to identify the viral particles when the clinical symptoms of enteritis are present, and it does not allow for the further discrimination and characterization of similar viruses, which require the combined use of PCR and sequencing. Based on these conclusions, presently, in routine laboratory procedures, all of the samples from turkey pullets showing enteritis are examined using IAEM with a 1:40 dilution of the original convalescent sera. Samples are simultaneously subjected to RT-PCR to identify the RV and define the Group (A, D, F or G) [38], as well as to identify and type the AstV [37].

**Table 4:** Number and type of multiple infections (association of two of more viruses) observed using IAEM.

Type of Association	1994	1995	1996	1997	1998	1999	2000	2001	2002	2003	2004	Tot.
Rotavirus + Astrovirus				1	7	7	6	10	4	17	4	56
Rotavirus + Astrovirus + Enterovirus-like										1		1
Rotavirus + Enterovirus-like		2		1	1		1	1		1	1	8
Rotavirus + Parvovirus-like							1		1	2		4
Rotavirus + Coronavirus										1		1
Astrovirus + Parvovirus-like								1	1			2
Astrovirus + Adenovirus									1		1	2
Astrovirus + Coronavirus										1		1
Enterovirus-like + Parvovirus-like					1					1		2
Adenovirus + Parvovirus-like							1			1		2
Total	0	2	0	2	9	7	9	12	7	25	6	79

### 3.3. Parvovirus-Like Particles in Pheasants

During the 1994 hatching season in northern Italy, a pathological syndrome was observed in three different pheasant (*Phasianus colchicus*) rearing facilities within a 20-km radius. The disease in 12 to 25-day-old pheasants was characterized by lethargy, anorexia, diarrhea, weight loss, sudden death and a high mortality (~50%). The affected pheasants had identical clinical signs and lesions, blood suffusions or small subcutaneous hemorrhagic areas and muscular scattered pinpoint hemorrhages, with pale livers that were mottled due to hemorrhagic suffusions, catarrhal duodenitis with swelling of the intestinal loops, and the spleen and bursa of Fabricius were frequently atrophied. Since the disease was not similar to any other previously reported in pheasants, several birds from each outbreak were referred to the diagnostic laboratory for necropsy at different times, and different pools of sera were collected from convalescent birds at two affected farms [39].

**Figure 6:**
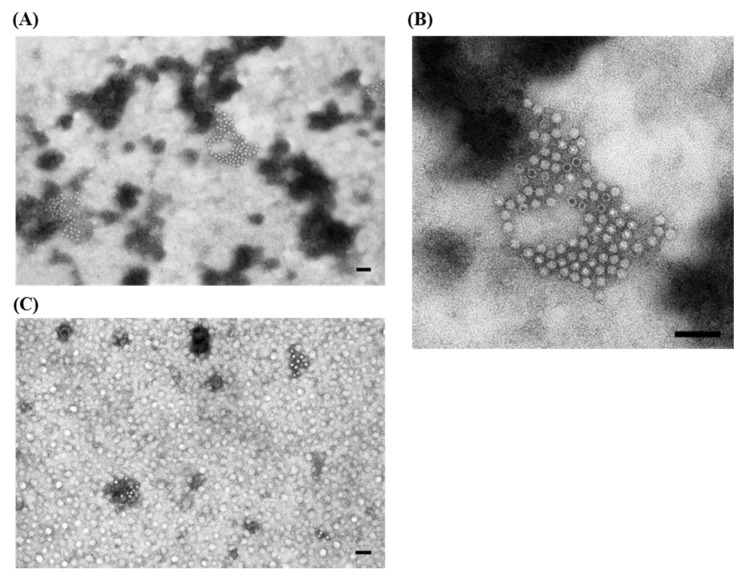
Electron micrograph of ultracentrifuged liver homogenate from a 20-day-old pheasant. (**A**) Aggregates of non-enveloped viral particles, 18–22 nm in diameter, morphologically corresponding to parvovirus, surrounded by fuzzy halos of antibodies are visible. Immuno-aggregation electron microscopy (IAEM) preparation with a pool of convalescent sera diluted 1:20; (**B**) Higher magnification of (**A**) showing the group of empty and full parvovirus-like particles; (**C**) IAEM negative control: single dispersed small viral particles are visible. Negative staining (2% sodium phosphotungstate). TEM Philips CM10, 80 kV. Bar = 100 nm.

An EM examination revealed small, non-enveloped icosahedral viral particles, ~18–22 nm in diameter, that morphologically resembling parvovirus and/or circovirus. Virions were present in large quantities, both as single particles and in small groups. IAEM examinations, particularly when using 1:20 sera dilution, revealed immunoaggregates composed of numerous virions surrounded by fuzzy halos of antibodies (Figure 6A,B). Virus-antibody aggregates were observed in samples from all three outbreaks using the two convalescent sera pools. Preformed aggregates were rarely observed in EM directly (Figure 6C).

Further attempts to characterize and precisely identify the taxonomy of such viruses were unsuccessful. The possibility that they were circovirus was excluded (Lavazza A. and Todd D., unpublished results) by using the same molecular approach that led to the first identification of the canary circovirus [40]. Unless their taxonomy can be stated, the definition “parvovirus-like virus” seems to be appropriate. With regard to diagnosis, no other methods have been developed and convalescent sera are still commonly applied to diagnose pheasant hepatitis, a disease that is almost regularly observed in game birds and farms every year during the hatching season.

### 3.4. Lagoviruses: Rabbit Hemorrhagic Disease Virus (RHDV) of Rabbits and European Brown Hare Syndrome Virus (EBHSV) of Hares

The genus Lagovirus is contained in the family Caliciviridae and includes virus species affecting only lagomorphs (rabbits and hares). RHDV, which was first described in China in 1984, causes a highly contagious and usually fatal (70%–90% mortality) disease of wild and domestic rabbits (*Oryctolagus cuniculus*) [41]. The gross lesions include hyperemia, congestion and diffuse hemorrhages, liver degeneration, necrosis and splenomegaly [42]. When RHD initially appeared in Europe, in late 1986, its etiology was not yet defined and different hypotheses, including intoxication and bacterial or viral infection, had been put forward. However, similarities to the existing EBHS described in brown hares (*Lepus europaeus*), which had been almost endemic in Europe since the early ’80s, became apparent. Unfortunately, the etiology of EBHS was still unclear [43].

**Figure 7:**
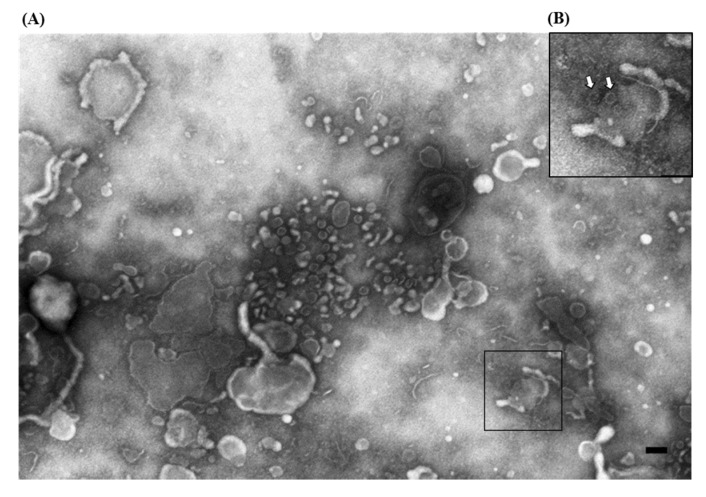
Electron micrograph of ultracentrifuged trachea and lung extract from rabbits infected with an organ homogenate of Rabbit Hemorrhagic Disease (RHD) from naturally infected rabbits. (**A**) A few rare calicivirus particles (see rectangle) are in the dirty and rich material, and thus, are hardly visible; (**B**) enlargement of inset, showing the typical morphology of calicivirus particles (⇧). The presence of RHDV particles in these pictures was done *ex post*. Thus, only after the definitive characterization of RHDV as a calicivirus were they recognized. Negative staining (2% sodium phosphotungstate). TEM Siemens Elmiskop 102, 80 kV. Bar = 100 nm.

**Figure 8:**
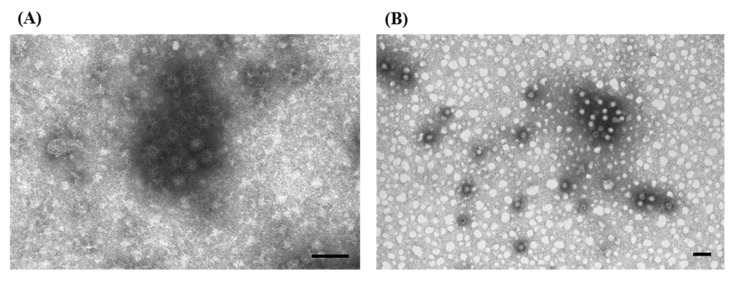
Electron micrograph of ultracentrifuged liver homogenate from rabbits naturally infected with Rabbit Hemorrhagic Disease. The empty icosahedral virions are ~30–32 nm with tiny, short external projections, surrounded by a dense halo of antibodies. (**A**) Immuno-aggregation electron microscopy (IAEM) preparation with a pool of convalescent sera diluted 1:40 from experimentally infected rabbits that survived the infection. Negative staining (2% sodium phosphotungstate). TEM Philips CM10, 80 kV. Bar = 100 nm; (**B**). IAEM negative control: RHDV particles are distributed singly or in small spontaneous groups.

**Figure 9:**
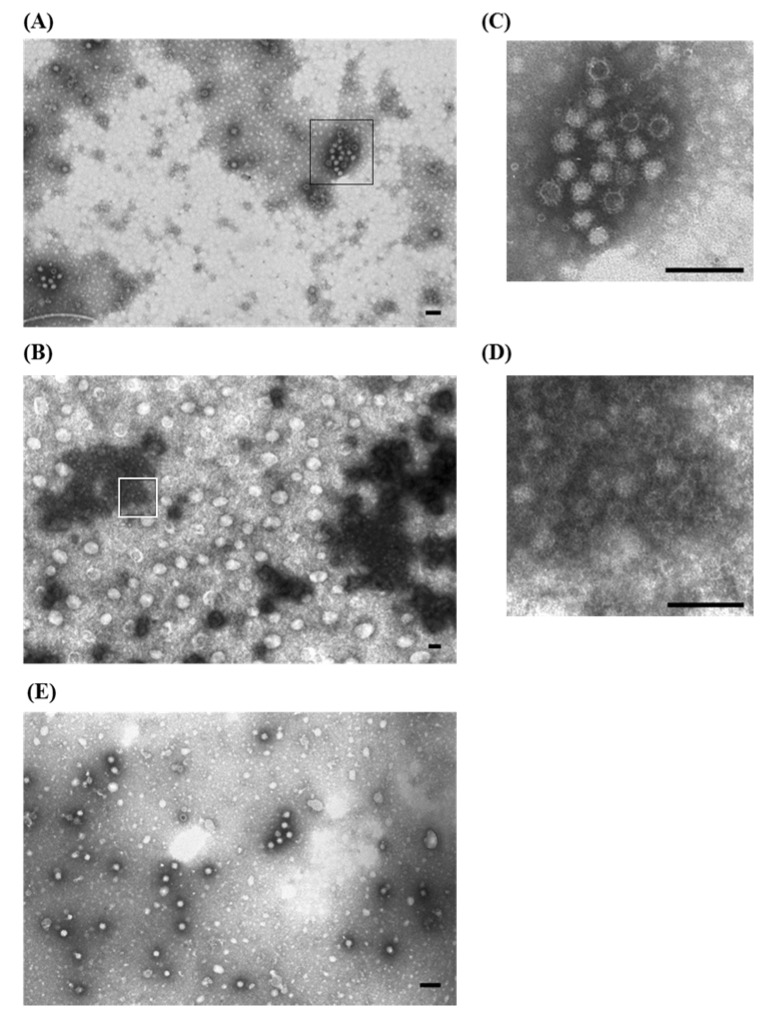
Electron micrograph of ultracentrifuged liver homogenate from European Brown Hare Syndrome (EBHS)-affected hares. Immuno-aggregation electron microscopy preparation with: (**A**) anti-Rabbit Hemorrhagic Disease (RHD) RHDV convalescent sera diluted 1:20; (**B**) anti-EBHSV homologous convalescent hare sera diluted 1:50; (**C**,**D**); enlargement of insets, showing the typical morphology of calicivirus particles. The size of aggregates, containing both empty and full particles, and the width of the antibody halo depend on the specificity of the convalescent sera used. Smaller and thinner halos were seen using the heterologous rabbit sera, and larger and thicker halos were seen using the convalescent hare sera; (**E**) IAEM negative control of EBHSV positive hare liver. Negative staining (2% sodium phosphotungstate). TEM Philips CM10, 80 kV. Bar = 100 nm.

The identification and the characterization of the viral agents of both diseases, RHDV and EBHSV, which are highly related but classified as phylogenetically distinct, was particularly challenging since the viral agents are difficult to isolate *in vitro* and there were severe lesions in non-target organs, such as the lungs and kidneys. We attempted to use ns-EM to identify the etiological agent (Figure 7A,B), but the existence of icosahedral virions of ~30–32 nm with tiny and short external projections, morphologically resembling calicivirus, was only obtained by IAEM using convalescent sera taken from animals that survived experimental infections. In fact, we observed compact groups of a variable number of viral units, surrounded by a dense halo of antibodies (Figure 8A) in most of the samples taken from animals showing typical signs and lesions [44,45]. By using both the anti-RHDV convalescent sera (Figure 9A) and homologous convalescent hare sera (Figure 9B) for IAEM examinations of liver homogenates of EBHS affected hares, we were able to identify the viral etiological agent (EBHSV) [45,46].

IAEM is no longer used as a routine diagnostic method for the detection of RHDV and EBHSV in clinical samples. In fact, after the initial definition of the viral etiology, many other diagnostic methods, which are more easily performed and applicable for screening surveys and antigenic typing (e.g., antigenic and antibody ELISAs), or have greater sensitivity and specificity (e.g., PCR methods), were developed and validated [47]. Nevertheless, the results obtained using IAEM with convalescent sera, as the first approach in the identification and diagnosis of lagoviruses, are valid proof of its importance and utility for discovering new animal viruses.

## 4. Conclusions

IAEM is a very sensitive and specific EM method. Hyperimmune serum and monoclonal antibodies may be used, but, based on our experience, IAEM with convalescent sera is a highly useful technique in the following situations: when an emerging or re-emerging virus is the etiological agent of an undiagnosed clinical outbreak; when the lack of immunological reagents and/or available primers may cause the failure of alternative diagnostic methods; when it is difficult to isolate virions *in vitro*; and when there is no indicative clinical suspect. The cases described in this paper are a clear demonstrations of such statements: IAEM with convalescent sera was used for (I) the first identification of lagoviruses, pig torovirus and pheasant parvovirus; (II) for an easier diagnosis of non-cultivable viruses, such as non-A rotavirus, turkey astrovirus and PED coronavirus; and (III) for detecting mixed infections, such as rotavirus RV associated with astrovirus in turkey or circovirus associated with enterovirus in pigs.

In terms of methodology, the most critical aspect of IAEM is the determination of the optimal dilution of sera, which induces the formation of immunoaggregates of adequate size with a thin halo of antibodies around the virions and a good conservation of the virion morphology. Indeed, the addition of specific controls to the IAEM reaction permits the validation and assessment of the immunoaggregation. The antibody titer in convalescent sera is usually not very high and, therefore, such sera usually work better at low dilutions (1:5 to 1:50).

However, there are some conditions that can limit the applicability of IAEM and affect obtaining satisfactory results. These include (I) mistakes in sampling (non-target organs); (II) samples taken during the chronic phase of a disease when the viral load is too low or a spontaneous immunoreaction has already occurred; (III) incorrect preservation of samples, causing the destruction and/or alteration of the virion’s morphology; and (IV) the subjectivity of examinations, which is related to the ability and experience of the operator. Nevertheless, some of these adverse conditions may affect other diagnostic techniques as well. Immunoassays may result in false negatives when sampling is not done properly (non-target organs, low viral load or chronic phase) or samples are not well preserved. Even very sensitive molecular methods (PCR) may also be affected by improper preservation of samples due to nucleic acid degradation.

The unique conditions that can limit the results obtained by IAEM are related to the subjectivity of the EM examination, which is highly dependent on the observer. However, the other “modern” technologies show some additional limitations. Antigen assays may fail to detect agents that differ in antigenic make up. Nucleic acid amplification techniques only detect genomic sequences that are known *a priori* and the PCR reaction may be inhibited by contaminants in the sample. Moreover, for a number of rare agents and, obviously, for the new ones, specific reagents are still lacking.

Apparently, there is no single method for the accurate and rapid laboratory diagnoses of all viral infections. Thus, when such a diagnosis is needed, ns-EM methods, and particularly IAEM, can be employed simultaneously with other front-line methods [6]. In fact, they permit the quick detection of non-cultivable viruses and multiple viral infections. In addition, thanks to the undirected “open view” of conventional TEM and its “catch all” property, emerging and new, or unrecognized, viruses may be identified.

Finally, as demonstrated by several studies that were inspired by our observations and identifications [11,12,15,16,17,22,23,37,40], the results obtained using ns-EM, including IAEM, as a diagnostic method may be the basis for further studies on the antigenic and molecular characterizations of viruses, their pathogenesis and epidemiology.
